# Synthesis and Characterization of Carboxymethyl Chitosan Nanosponges with Cyclodextrin Blends for Drug Solubility Improvement

**DOI:** 10.3390/gels8010055

**Published:** 2022-01-12

**Authors:** Syeda Sadia Batool Rizvi, Naveed Akhtar, Muhammad Usman Minhas, Arshad Mahmood, Kifayat Ullah Khan

**Affiliations:** 1 Department of Pharmaceutics, Faculty of Pharmacy, The Islamia University of Bahawalpur, Bahawalpur 63100, Punjab, Pakistan; sadiarizvi1214@gmail.com (S.S.B.R.); naveed.akhtar@iub.edu.pk (N.A.); 2 College of Pharmacy, University of Sargodha, University Road, Sargodha 40100, Punjab, Pakistan; 3 College of Pharmacy, Al Ain University, Abu Dhabi Campus, Abu Dhabi 112612, United Arab Emirates; arshad.mahmood@aau.ac.ae; 4 Quaid-e-Azam College of Pharmacy, Sahiwal 57000, Punjab, Pakistan; kifayat.rph@yahoo.com

**Keywords:** carboxymethyl chitosan (CMC), β-cyclodextrin, solubility enhancement, nanosponges, docetaxel, cyto-toxicity

## Abstract

This study aimed to enhance the solubility and release characteristics of docetaxel by synthesizing highly porous and stimuli responsive nanosponges, a nano-version of hydrogels with the additional qualities of both hydrogels and nano-systems. Nanosponges were prepared by the free radical polymerization technique and characterized by their solubilization efficiency, swelling studies, sol-gel studies, percentage entrapment efficiency, drug loading, FTIR, PXRD, TGA, DSC, SEM, zeta sizer and in vitro dissolution studies. In vivo toxicity study was conducted to assess the safety of the oral administration of prepared nanosponges. FTIR, TGA and DSC studies confirmed the successful grafting of components into the stable nano-polymeric network. A porous and sponge-like structure was visualized through SEM images. The particle size of the optimized formulation was observed in the range of 195 ± 3 nm. The fabricated nanosponges noticeably enhanced the drug loading and solubilization efficiency of docetaxel in aqueous media. The drug release of fabricated nanosponges was significantly higher at pH 6.8 as compared to pH 1.2 and 4.5. An acute oral toxicity study endorsed the safety of the system. Due to an efficient preparation technique, as well as its enhanced solubility, excellent physicochemical properties, improved dissolution and non-toxic nature, nanosponges could be an efficient and a promising approach for the oral delivery of poorly soluble drugs.

## 1. Introduction

Docetaxel (DTX), a semi-synthetic toxoid, is a potent anti-neoplastic drug that is approved for the treatment of prostate, breast, head and neck cancers, as well as lung carcinoma [1]. Docetaxel is an extensively used, anti-cancerous drug whose antitumor efficacy is accredited to its ability to disrupt the normal process of microtubule assembly and disassembly, followed by cell apoptosis and death [2,3]. The clinical application of DTX is hindered by its poor aqueous solubility and low oral bioavailability [4]. Therefore, there is a requisite for the fabrication of a new drug delivery system for the efficient loading and release of the poorly water-soluble drug DTX, in order to boost its therapeutic efficacy and to minimize its adverse effects.

Cyclodextrins are cyclic oligosaccharides consisting of a-1,4-linked cyclic glucopyranose oligomers synthesized by the enzymatic degradation of starch [5,6,7]. Cyclodextrins are of three types, called α, β and γ, comprising six, seven and eight glucopyranose units, respectively [8]. Among these types, β cyclodextrin is the most frequently used, as it is easily accessible, less irritating and the most economical cyclodextrin [7]. They are frequently utilized as “molecular cages” in the pharmaceutical, food and cosmetic industries [9]. In the pharmaceutical industry, these fundamental properties can improve the solubility, dissolution profile, and bioavailability of hydrophobic drug molecules [10,11].

Carboxymethyl chitosan is an attractive amphoteric polyelectrolyte containing both positive and negative charges. Basically, CMC is a hydrophilic derivative of chitosan [12] in which some OH groups of chitosan were substituted by CH_2_COOH groups [13]. Carboxymethyl chitosan not only exhibits excellent aqueous solubility but also possesses unique physical, chemical and biological properties such as biocompatibility, biodegradability, bio-adhesivity, bio-renewability, non-toxicity, high viscosity, large hydrodynamic volume and a good ability to form films, fibers and hydrogels [12,13]. Owing to these properties, it has been extensively utilized in various biomedical fields, e.g., as a bactericide, antitumor, antifungal [14], wound-dressing agent, moisture retention agent, bio-imaging, tissue engineering, blood coagulant and a polymer in different drug-delivery matrices [12,15].

2-Acrylamido-2-methylpropane sulfonic acid (AMPS) is a white crystalline powder and highly hydrophilic acrylic monomer. It is an acidic polymer that acts as a polyelectrolyte, which exhibits good thermal stability and pH-independent swelling behavior. Owing to its highly hydrophilic nature, high swelling and absorbing abilities, as well as its bio-compatibility, thermal stability and cohesion strength, it plays a vital role in the solubility enhancement of poorly soluble drugs [16,17]. *N,N*’-Methylene bisacrylamide (MBA) is a bi-functional monomer with two identical unsaturated double bonds, which is widely applied as cross-linking agent in various drug delivery systems. MBA-based formulations exhibit multiple functionalities, including an improved swelling and release profile. For this reason, they are preferred over other cross-linkers [18].

Nanosponges are actually nano-sized hydrogels with tiny sponges ranging 20–200 nm, which can be filled with a wide variety of drugs [19]. These nano-sized tiny sponges can circulate around the body until they encounter the specific target site, at which point they stick onto the surface and begin to release the drug in a controlled and predictable manner. As the drug can be released at the specific target site instead of circulating throughout the body, it will be more efficacious for a particular dosage. Another idiosyncratic property of these sponges is their aqueous solubility due to their porous, spongy and non-collapsible structure. This encapsulating type of nanoparticle actually encapsulates the drug molecules within its core, which could revolutionize the treatment of many diseases, and early trials suggest that this technology is up to five times more effective at delivering drugs for breast cancer compared to conventional methods [20,21].

Owing to the poor aqueous solubility and low oral bioavailability of DTX, oral administration represents a great challenge in DTX delivery [22,23]. Within this context, it is recommended to first prepare the bi-polymeric nanosponges loaded with DTX, to meet the oral administration requirements of DTX. To date, a number of research works have been reported on DTX/CD nanoparticles [24,25,26]. However, there is no specific research on βCD-CMC-g-poly (AMPS)-based nanosponges loaded with the poorly soluble drug, DTX.

The aim of the present study was to investigate βCD-CMC-g-poly (AMPS)-based nanosponges, a nano-version of hydrogels, as a potential drug carrier to enhance the aqueous solubility and dissolution rate of docetaxel. Firstly, drug loading and solubilization efficiency were determined. Then, fabricated βCD-CMC-g-poly (AMPS) nanosponges were characterized by various analytical techniques and evaluated for in vitro drug release. In vivo toxicity studies were also performed to confirm the safety of the fabricated nanosponges.

## 2. Results and Discussion

### 2.1. FTIR Analysis of Nanosponges

**Figure 1:**
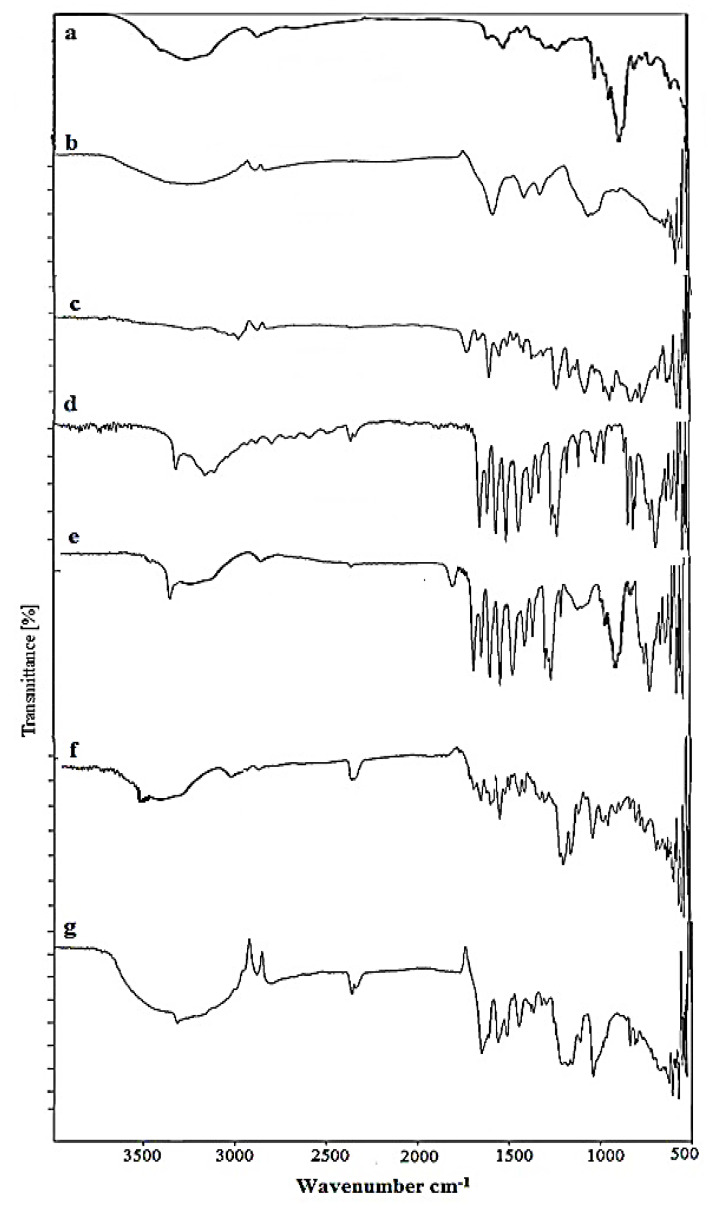
FTIR spectra of (**a**) pure drug docetaxel (DTX), (**b**) β-CD (**c**) Carboxymethyl chitosan (CMC), (**d**) AMPS, (**e**) physical mixture, (**f**) DTX loaded βCD-co-poly (AMPS), and (**g**) DTX loaded βCD-CMC-co-poly (AMPS) nanosponges.

Fourier transform infrared (FT-IR) spectroscopy is an analytical technique used to ascertain the presence of a functional group, extent of cross-linking and structural changes that occurred during the polymerization process. The FTIR spectra of each constituent, as well as the fabricated product, i.e., nanosponges, were recorded as shown in Figure 1. The FTIR spectrum of Docetaxel showed characteristic peaks at 3481 cm^−1^ for N-H stretching and 3337.96 cm^−1^ for O-H stretching. Short peaks at 1733.48 cm^−1^ and 1716.85 cm^−1^ may be ascribed to C=O stretching vibration. Another peak at 1624.37 cm^−1^ is attributed to N-H plane bending [27].

In the FTIR spectrum of β-Cyclodextrin, a broad transmittance peak is reported at 3292 cm^−1^ which is due to the valence vibrations of O-H bonds in the primary hydroxyl groups (C-OH). The IR spectrum of β-cyclodextrin also reported an absorption peak with a maximum at 2927.82 cm^−1^ due to vibrations in C-H bonds hosted in –CH and –CH2 groups, at 1632.66 cm^−1^ due to H-O-H bending, at 1152.74 cm^−1^ due to the C-O stretch of cyclohexane ring, and at 1021 cm^−1^ to 1077 cm^−1^ due to vibrations in the C-O bonds in ether and hydroxyl groups. [28,29]. All recorded FTIR peaks for β-Cyclodextrin are shown in Figure 1.

The IR spectra of pure Carboxymethyl chitosan showed a characteristic broad band at 3260.05 cm^−1^ due to –OH and N-H amine symmetrical stretching vibrations [30]. Two short peaks at 2885.91 cm^−1^ and 2826.45 cm^−1^ correspond to C–H stretching vibrations [31]. A sharp peak appeared at 1579.01 cm^−1^ due to the N-H bending of primary amines [32], while the peak at 1407.73 cm^−1^ corresponds to C-N stretching vibrations. The characteristic strong peak at 1320.28 cm^−1^ is attributed to C-O stretching vibration. A similar peak was reported in the FTIR spectra of carboxymethyl-chitosan-based nanocarriers [30]. Another peak appeared at 1053.21 cm^−1^, which represents the C-O stretch of –CH_2_–OH in primary alcohols.

The FT-IR spectrum of pure AMPS revealed peaks at 2986 cm^−1^, representing the C-H stretching of –CH_2_, while two bands at around 1666 cm^−1^ and 1612 cm^−1^ were due to C=O stretching and C=C stretching, respectively [33]. A peak at 1550.37 cm^−1^ represents secondary amide N-H deformation, while the C-H bending peak of AMPS was observed at 1417.72 cm^−1^. The two peaks observed at 1372.13 cm^−1^ and 1126.25 cm^−1^ indicate S=O stretching due to the presence of SO_3_H group in AMPS. A strong absorption band in the region 1076 cm^−1^ to 941 cm^−1^ indicated the S–O–C group [34].

No interaction was revealed when the contents were physically mixed. The major peaks that were present in IR spectrum of βCD, CMC, DTX and AMPS were also present in the IR spectrum of a physical mixture, which indicates a lack of interaction among excipients. No changes in the peak’s shape, intensity and position were reported for the physical mixture. When the aforementioned IR spectra were compared with the IR spectrum of fabricated βCD-co-poly (AMPS) nanosponges, the results indicated the successful cross-linking of β-cyclodextrin with the monomer. The reported peaks in the individual β-cyclodextrin and AMPS were slightly shifted and the intensity of the peaks was also modified.

Furthermore, the IR spectra of DTX-loaded βCD-CMC-co-poly (AMPS) and DTX loaded βCD-co-poly (AMPS) nanosponges markedly differed from the spectra of physical mixture and pure ingredients, as shown in Figure 1. It was observed that all the characteristic peaks in drug (DTX) were present (Figure 1a,g), with slight modifications. The reduction in peak intensity and height, as well as the slight shifting/modification and disappearance/emergence of peaks in the spectra of loaded formulations, indicated the complex formation between polymers (CMC and β-CD) and monomer (AMPS), as well as the incorporation of this drug into the system.

### 2.2. Powder X-ray Diffraction Analysis

**Figure 2:**
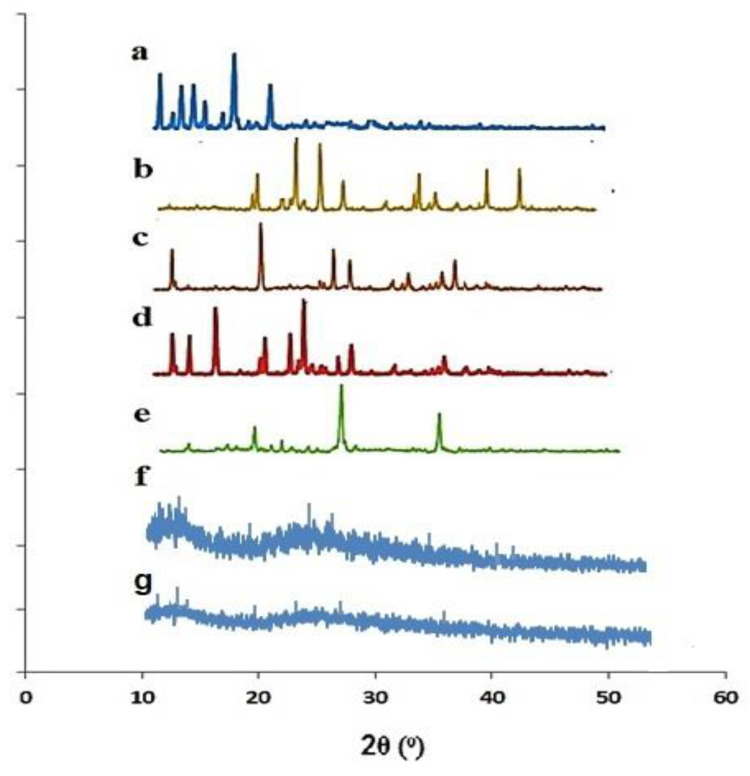
PXRD patterns of (**a**) pure drug docetaxel (DTX), **b** β-CD, (**c**) Carboxymethyl chitosan (CMC), (**d**) AMPS, (**e**) physical mixture, (**f**) DTX loaded βCD-co-poly (AMPS), and (**g**) DTX loaded βCD-CMC-co-poly (AMPS) nanosponges.

A PXRD diffractogram of Docetaxel, β-CD, CMC, AMPS, physical mixture, and fabricated nanosponges was carried out to determine amorphous or crystalline behavior as illustrated in Figure 2. A crystalline nature prohibits the solubility of a substance, while amorphous natures show higher intermolecular energy levels and higher molecular mobility, thus providing a better platform for improving the solubility and dissolution rate [35].

PXRD diffractogram of docetaxel revealed blunt and prominent peaks at 2 θ = 10.21°, 11.6°, 12.5, 15.01°, 15.7° and 19.01° elucidating its crystallinity [36]. Similarly, a PXRD diffractogram of β-CD exhibited sharp and intense peaks at 2Ɵ = 20.56°, 23.97°, 27.91°, 34.87°,40.39° and 44.10° [37]. These peaks proved the crystalline nature of β-CD. In another study, an XRD study of pure β-CD was performed, and their findings regarding the crystalline properties of sample were similar to our findings [28]. However, in the case of pure Carboxymethyl chitosan, crystallinity was proved by the peaks witnessed at 2θ = 10.1°, 19.78°, 27.11°, 28.71°, 32.15°, 34.33°, and 36.98°. Previously, peaks of a similar intensity were reported in PXRD analysis [31,38] Moreover, the crystallinity of AMPS was evident by the presence of characteristics peaks at 2θ = 11.52°, 13.10°, 15.80°, 20.31°, 22.11°, 23.10°, 26.55° and 27.71° [39]. Fewer, but sharp, peaks were observed in a PXRD diffractogram of a physical mixture of drug and polymer at 2Ɵ = 19.21°, 28.11° and 36.91°, indicating that the crystalline nature partially declined. In the case of an XRD diffractogram of DTX-loaded βCD-co-poly (AMPS) and DTX-loaded βCD-CMC-co-poly (AMPS) nanosponges, the sharp characteristic peaks in pure drug DTX were noticeably masked by the amorphous system of nanosponges, which is more soluble than the crystalline form, as shown in Figure 2f,g. Previously, Khan et al. also reported on the solubility enhancement of poorly soluble drug olanzapine by the amorphous system of nanomatrices [40].

### 2.3. TGA Analysis of Nanosponges

**Figure 3:**
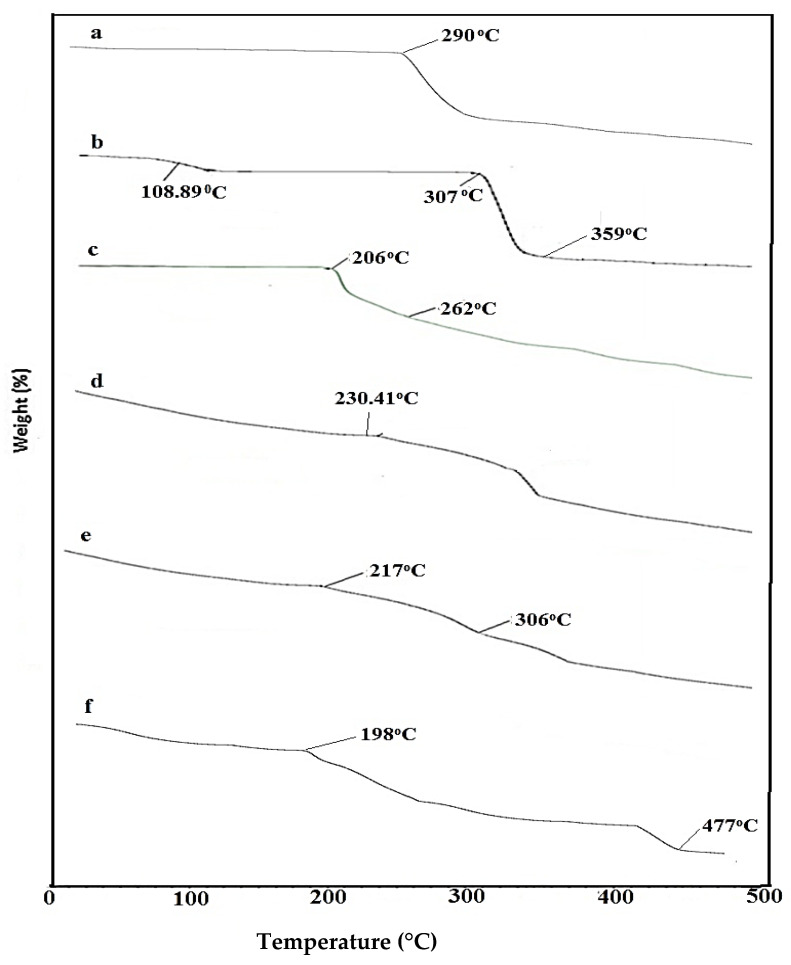
TGA thermogram of (**a**) Carboxymethyl chitosan (CMC), (**b**) β-CD, (**c**) AMPS, (**d**) physical mixture, (**e**) βCD-co-poly. (AMPS), and (**f**) βCD-CMC-co-poly (AMPS) nanosponges.

A TGA thermogram of pure β-CD showed thermal degradation in three steps, as shown in Figure 3: First, prominent weight loss, i.e., 13.03% was observed at 108.88 °C due to the loss of water contents from β-CD cavities; Second, prominent weight loss, i.e., 18.35% was observed at 307.46 °C representing the melting point of β-CD. The main decomposition, i.e., 84.28% was observed at a higher temperature of 359 °C owing to degradation of the residual polymer. CMC presented maximum thermal decomposition at 290 °C with a weight loss of 26.82% due to the decomposition of the carboxymethyl group [41]. A TGA thermogram of AMPS revealed thermal degradation in two steps, which occurred at 206 °C due to loss of moisture content, with a corresponding weight loss of 2.34% and at 262 °C due to combustion above the melting point with a weight loss of 48.84% [42]. In case of a physical mixture, weight loss of more than 80% was observed at 230.41 °C, indicating that there is less stability and no complexation among physically mixed excipients. The βCD-co-poly (AMPS) formulation showed an initial weight loss of 16.19% at 217 °C due to the breakdown of sulphonic acid functional groups from polymeric backbone, followed by a decomposition event that started at 306 °C with weight loss of 40.67%. The βCD-CMC-co-poly (AMPS) formulation also exhibited a two-step decomposition phase with an initial decay in the thermogram at 198 °C due to moisture loss and a major decay at 477 °C, i.e., 39.89%, representing the degradation of residual mass. In comparison, it was observed that the βCD-CMC-co-poly (AMPS) formulation (477 °C) exhibited higher thermal stability than that of the βCD-co-poly (AMPS) formulation.

### 2.4. DSC Analysis of Nanosponges

**Figure 4:**
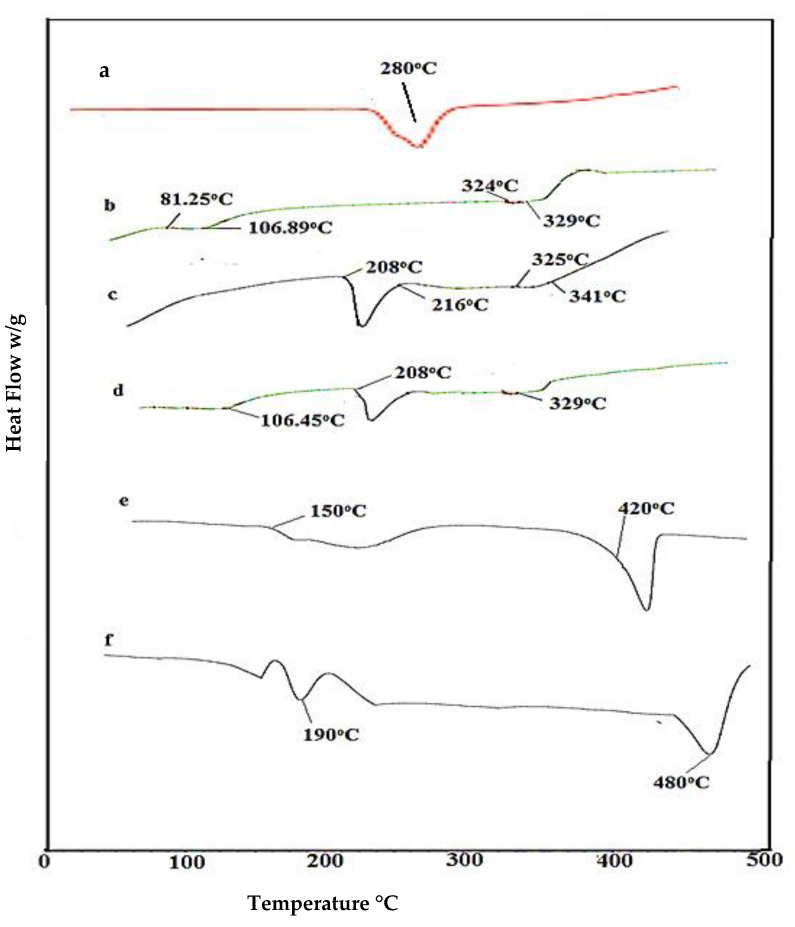
DSC thermogram of (**a**) Carboxymethyl chitosan (CMC), (**b**) β-CD, (**c**) AMPS, (**d**) physical mixture, (**e**) βCD-co-. Poly (AMPS), and (**f)** βCD-CMC-co-poly (AMPS) nanosponges.

DSC is a thermal analysis technique capable of determining first- and second-order thermal transitions, such as melting (Tm), crystallization (Tc) and glass transition (Tg) phenomena. DSC thermograms of β-Cyclodextrin, CMC, AMPS, physical mixture and fabricated nanosponges are shown in the Figure 4. In the DSC curve investigation of β-CD, two characteristic sharp endothermic peaks appeared, the first at 106.89 °C and second at 329 °C [43]. The DSC curve of CMC indicated an initial endothermic peak at 280 °C owing to water loss. This evaporation of water in the endothermic peak indicates the physical or molecular changes in carboxymethylation [44,45]. Moreover, the DSC thermogram of AMPS presented its first endothermic peak at 208 °C, indicating its melting point, followed by a second peak at 216 °C, indicating its decomposition [46,47]. In the DSC thermogram of a physical mixture, strong endothermic peaks of polymer and monomer appeared at 106.45 °C, 208 °C and 329 °C, thus proving the lack of interaction among excipients and lower stability over a wider temperature range. The βCD-co-poly (AMPS) spectra showed its first endothermic peak at 150 °C, representing the melting of formulation, and its second peak at 420 °C, indicating decomposition. In this study, it was observed that the DSC spectra of βCD-CMC g-poly (AMPS) nanosponges were markedly different from βCD g-poly (AMPS), polymers, and the monomer thermogram. The characteristic sharp endothermic peaks at 106.89 °C (βCD), 280 °C (CMC), and 216 °C (AMPS) completely disappeared in the DSC curve of fabricated βCD-CMC g-poly (AMPS) nanosponges. The DSC curve of βCD-CMC g-poly (AMPS) nanosponges first showed an endothermic peak at 190 °C, indicating the melting of formulation, followed by a decomposition peak at 480 °C, which ensured the successful formation of new polymeric network with improved thermal stability. Recently, Asghar et al. prepared spongy matrices of acyclovir with β-CD and HPMC [46]. The formulation’s stability was promoted due to the complexation of bi-polymers and monomer, as in our findings. Overall, the results of DSC and TGA studies proved the successful formation of bi-polymeric nanosponges and enhanced thermal stability.

### 2.5. Scanning Electron Microscopy (SEM)

**Figure 5:**
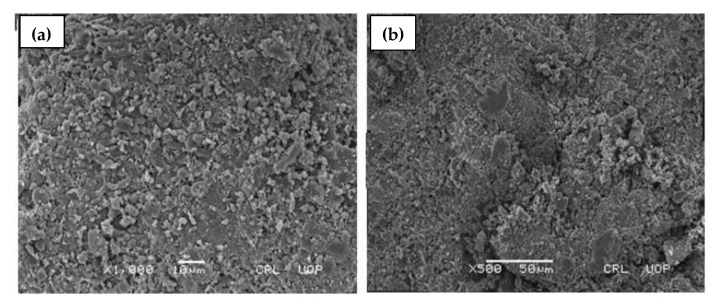
SEM (scanning electron microscopy) micrographs (**a**) βCD-CMC-g-poly (AMPS), (**b**) βCD-g-poly (AMPS) nanosponges.

To determine the surface morphology of the developed βCD-CMC-g-poly (AMPS) and βCD-g-poly (AMPS) nanosponges, SEM micrographs were taken using a scanning electron microscope (SEM) at different magnifications, as presented in Figure 5a,b, respectively. SEM micrographs of the developed nanosponges clearly revealed that these nanosponges were more compact, highly porous, and contained some white texture. The numerous pores present in the system facilitated the accelerated influx and rapid uptake of aqueous media, resulting in rapid swelling and drug release from the polymeric network [28,48]. The previous reported studies on cyclodextrin-based nanosponges presented similar findings [40,46,49].

### 2.6. Particle Size Analysis

The size of nanosponges plays a significant role in their dissolution, solubility, and, ultimately, drug release from carrier system. According to the Ostwald–Freundlich equation, solubility is directly proportional to particle size. The smaller the particle, the greater its dissolution and solubility, as a smaller particle size provides a large surface area, which in turn enhances the dissolution rate and improves the bioavailability of the drug [50]. Particle size analysis of the nanosponges was characterized by zeta sizer (Malvern Instruments, Malvern, UK). The average particle sizes of the developed nanosponges (F-0 to F-9) are presented in Table 1. The polydispersity index (PDI) value was found to be 0.279. The small PDI value indicated that developed nanosponges have low affinity to form aggregates. Hecq and Deelers et al. fabricated nanocrystals for a solubility enhancement of Nifedipine and reported that a reduction in particle size resulted in enhanced solubility and rapid release of the drug [51]. Similarly, Kifayat and Faisal et al. fabricated nanogels and nanomatrices to improve the solubility of a poorly water-soluble drug olanzapine and chlorthalidone, respectively [52,53].

### 2.7. Drug Entrapment Efficiency (DEE %) and Drug Loaded contents (DLC%)

A post loading technique was utilized to load docetaxel in fabricated nanosponges. The extent of cross-linking, as well as the swelling behavior, of the cross-linked network greatly influences the % DEE and Loaded contents. The drug entrapment efficiency (%) and drug-loaded contents (%) of all developed formulations are reported in Table 2. All the results were found to be within the acceptable limit, i.e., 85.32–87.4% and 89.32–91.74%. All nanosponges showed more than 70% drug loading. Formulations F-8 and F-9 presented minimum drug entrapment efficiency and drug-loaded contents, i.e., 71.40–74.44% and 80.43–83.62%. This attributed to the increased concentration of the cross-linker and the highly cross-linked and dense polymeric network; as a result, the penetration of solvent into this highly cross-linked and dense mass is hindered. Badshah et al. reported the same findings: increasing the cross-linker’s concentration results in poor drug entrapment efficiency [53]. On the other hand, maximum drug entrapment efficiency was indicated by formulations with an increased concentration of polymer and monomer (F-1 to F-6), which might be attributed to the high swelling ability of bi-polymeric nanosponges, resulting in an increase in channeling owing to the repulsion of more sulfonic acid (–SO3) groups within the bi-polymeric spongy network and the formation of an inclusion complex [28]. It was also observed that the drug entrapment efficiency (%) was dramatically increased by utilizing bi-polymers (CMC, β-CD) as compared to single polymer (F-0 formulation), as presented in Table 2, which enhances the capacity of this spongy nanostructure to entrap the drug within the system. Yuklu et al. fabricated lansoprazole-loaded nanosponges by the emulsion solvent diffusion method by utilizing ethylcellulose, PVA and pluronic F-68 and dichloromethane as a solvent. In contrast to our findings, lansoprazole-loaded nanosponges showed decreased DLC % with an increasing polymer concentration due to the sticky nature of the product. Moreover, the drug entrapment efficiency also decreased with increasing polymer concentration due to the poor aqueous solubility of polymer [54].

### 2.8. Solubilization Efficiency 

**Figure 6:**
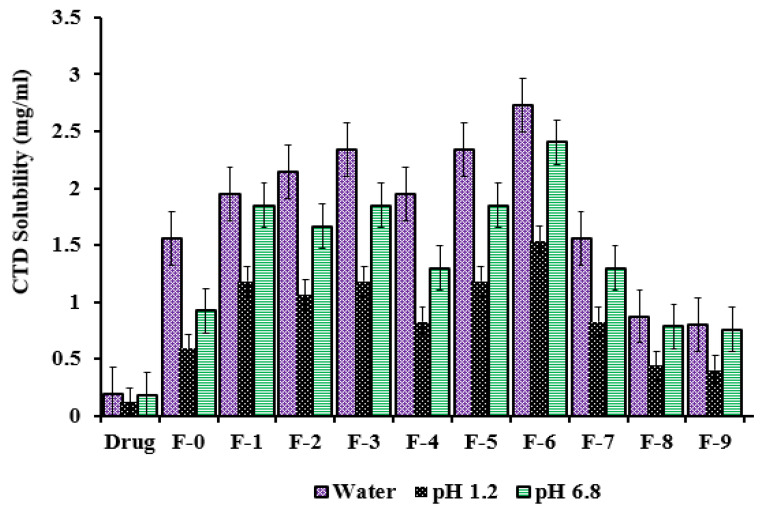
Solubility profiles of drug and fabricated nanosponges in water, pH 1.2 and 6.8.

The solubilization efficiency of all formulations was tested in distilled water and buffers of pH 1.2 and pH 6.8. This study was performed for the drug alone, and that of the nanosponges. Since docetaxel is a strongly acidic drug, the ionization of the drug will be higher in pH 6.8 and water as compared to that of pH 1.2. A noticeable difference was observed between the solubility profiles of nanosponges and that of the drug alone, as the solubility results of nanosponges were quite satisfying. There was a maximum 14-fold increase in the solubilization efficiency of the drug compared to that of the nanosponges, as shown in Figure 6. This 14-fold increase in the solubilization efficiency of the drug was observed in formulation F-6, in which the maximum concentration of monomer was used. Furthermore, the increase in the solubility profile of nanosponges was found t be higher in water compared to pH 1.2 and 6.8. This difference may be attributed to the pKa value of the drug. Since the pKa value of the drug is 10.9, its ionization will be higher in aqueous medium as compared to that of buffers of pH 1.2 and 6.8. Similarly, the solubility profiles of nanosponges in pH 6.8 were also higher compared to that of pH 1.2.

The mechanism involved in solubility enhancement can be elucidated on the basis of the structural features of β-CD. The internal cavity of the β-CD is highly lipophilic, while the external surface is hydrophilic. Whenever any drug is loaded in a β-CD-based drug delivery system, the hydrophobic abilities of the drug are mostly restrained by the internal lipophilic cavity of the polymer, whereas the hydrophilic abilities remain exposed to the external environment; hence, the hydrophilicity of the drug is enhanced and the maximum drug concentration is solubilized in the respective media [55]. Furthermore, the hydroxyl and amino groups of carboxy methyl chitosan result in significant protonation in neutral solution and increase carboxy methyl chitosan’s solubility in neutral and basic solutions without influencing other important characteristics. The solubility enhancement by fabricated bi-polymeric nanosponges may also be attributed to the amorphousness of docetaxel within a bi-polymeric system, enhanced docetaxel wettability, increased surface area, reduced particle size and solubilizing effect of fundamental hydrophilic excipients (β-CD, CMC, AMPS) [56]. The results revealed that the prime objective of fabricating nanosponges was fulfilled i.e., solubility enhancement of docetaxel. Previous studies also reported nanosponges as providing the best solubility enhancement for lipophilic drugs [57,58,59]. Furthermore, compared to other conventional βCD nanoparticles, our study depicted a marked increase in drug solubility at pH 6.8 [60].

### 2.9. Sol Gel Analysis

**Figure 7:**
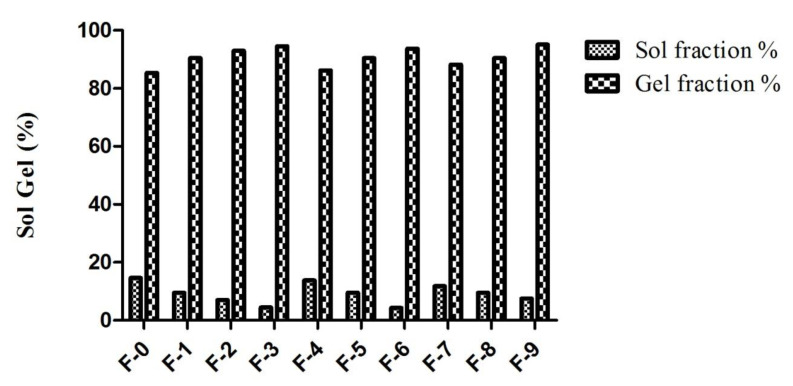
Sol-gel fraction of all synthesized nanosponges.

Sol gel analysis was executed to determine the un-crosslinked polymer fraction in a fabricated polymeric network using the Soxhlet extraction technique. The sol gel fraction of all fabricated formulations was obtained to appraise the influence of increasing polymer, monomer and cross-linker contents on gel%, as presented in Figure 7. The sol fraction demonstrates the amounts of uncross-linked polymer and monomer, which are solubilized during the Soxhlet extraction technique, while the gel fraction depicts the amounts of cross-linked polymer and monomer and is the insoluble fraction. The greater the gel fraction, the smaller the sol-fraction and vice versa. The results showed that the gel fraction was significantly enhanced by enhancing the ratio of β-CD, CMC, AMPS and MBA. An increased gel fraction indicates increasing polymer and monomer quantities. The reason for this is that increasing the polymer and monomer contents provides more free active sites for free radical polymerization reactions, thus resulting in a stable gel [61,62]. Nanosponges with an increase in cross-linker (MBA) concentration also led to the increased binding of monomer molecules to cross-linker molecules, forming compact networks [63,64]. Previously, similar results were also reported in a study where increasing the MBA concentration resulted in an increased gel fraction due to the increased availability free active sites to complete the reaction [65,66].

### 2.10. Swelling Analysis

**Figure 8:**
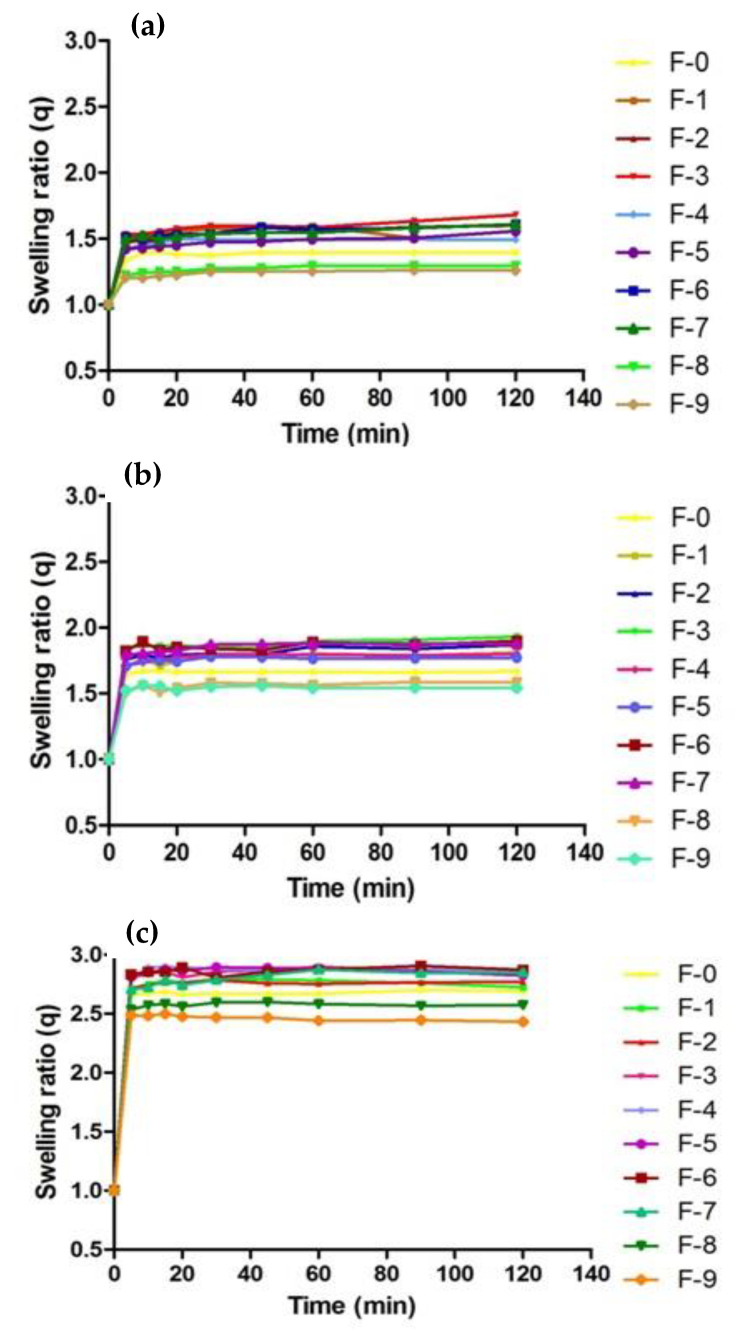
(**a**) Swelling ratio of developed nanosponges (F0–F9), in buffer solution of pH 1.2, (**b**) pH 4.5, and (**c**) pH 6.8.

Figure 8 represents the swelling analysis of developed nanosponges. A swelling analysis is important to determine the water-absorbing capacity and permeability of nanosponges [67], which are good determinants for drug release [68] from developed formulation, since they are exposed to biological fluid [69]. The swelling ratio of nanosponges was assessed at pH 1.2, 4.5 and 6.8. βCD-CMC-co-poly (AMPS)-developed nanosponges revealed a significantly high swelling at all pH levels. Abrupt swelling behavior was noticed within 5 min, which might be attributed to the spongy nature, nano-size and larger surface area of nanosponges. The developed nanosponges attained equilibrium swelling within 20–30 min. The overall swelling capacity of nanosponges at pH 6.8 was slightly greater as compared to that of pH 4.5 and pH 1.2 [43,60]. CMC contains carboxyl groups in the polymeric network, which exhibit more swelling in a basic medium than an acidic medium. This could be attributed to the fact that, in a basic environment, electrostatic repulsion between carboxylate anions (COO−) and the osmotic swelling force within polymeric nanostructure controls the expansion of the latter and, ultimately, the swelling behavior of nanosponges [41].

Furthermore, these carboxylate anions (COO−) have a strong tendency towards solvation compared to non-ionic groups in aqueous or alkaline media. As a result, an increased swelling of fabricated nanosponges has been observed at a basic pH. The addition of a second polymer i.e., CMC with β-CD, enhanced the swelling behavior of nanosponges as compared to β-CD-co-poly (AMPS) formulation; this increase in swelling might be associated with the highly porous and hydrophilic nature of β-CD, which generates more free active sites for the grafting of a monomer. Usually, the single polymeric formulation exhibits poor swelling/de-swelling properties, which can be boosted by the fabrication of a bi-polymeric network system [70]. Similarly, the nature and concentration of the monomer used has a profound effect on the swelling properties of the prepared formulation.

By increasing the AMPS concentration in fabricated nanosponges, a marvelous increase in swelling behavior was observed due to the increased number of sulphonic groups [71]. An AMPS is a hydrophilic monomer with ionic and non-ionic functional groups. As the number of ionizable groups (–SO3) increases due to dissociation, this promotes the swelling capacity of nanosponges due to repulsion among ionized species in the spongy network. In a previous study, similar findings were reported, in which the swelling capacity of the polymeric network was enhanced with the increase in AMPS concentration [72,73]. In contrast, by increasing MBA concentration in fabricated nanosponges, a noticeable decrease in swelling behavior was observed owing to the enhancement of cross-linking density and reduction in the flexibility of polymeric chains, which restricts their water-absorbing capacity and, ultimately, the swelling dynamics of nanosponges [64]. Previously, similar results were reported in the literature, showing reduced swelling with an increasing MBA concentration [74,75,76].

### 2.11. In Vitro Drug Release Studies

**Figure 9:**
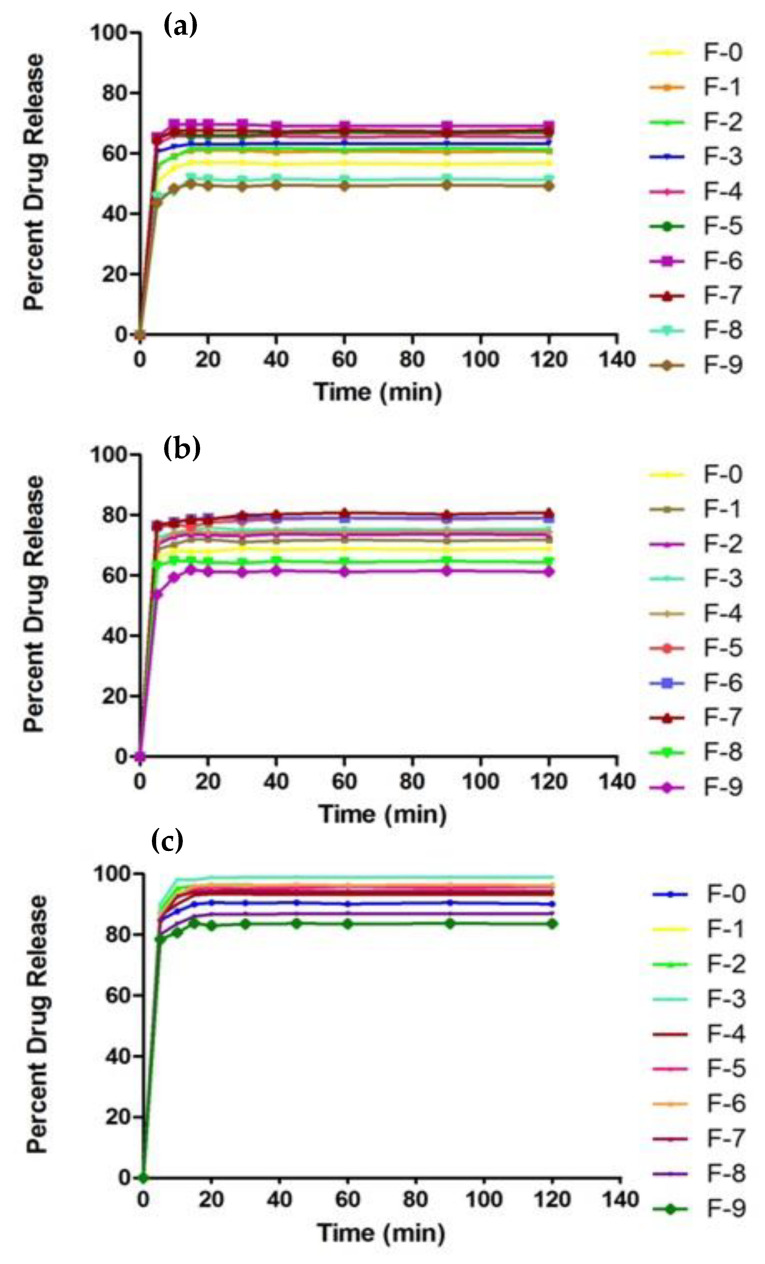
Drug release profiles of formulated nanosponges in buffer solution of (**a**) pH 1.2, (**b**) pH 4.5 and (**c**) pH 6.8.

To evaluate the drug release profile, in vitro drug release studies were executed for all the formulated nanosponges in three different mediums of pH 6.8, pH 4.5 and pH 1.2, as shown in Figure 9. The overall drug release from nanosponges at pH 6.8 was greater compared to that of pH 4.5 and pH 1.2. This rapid release of the drug in pH 6.8 may be due to the acidic nature of docetaxel. Due to the drug’s acidic nature, it becomes more ionized in a basic medium compared to acidic medium. Due to this greater ionization, the drug release and solubilization in basic medium (pH 6.8) is rapid. An abrupt release was noticed within 5 min, which attained equilibrium within 20–30 min. This can be attributed to the hydrophilic and spongy nature of the developed system, which facilitated the rapid influx of water, resulting in the increased swelling of nanosponges at a higher pH and, ultimately, an increased in drug release from the polymeric network. This quick responsive behavior of nanosponges differentiates it from other polymeric systems fabricated for the purpose of solubility enhancement. Rapid dissolution may also be reinforced by a small particle size, increase in surface area, decrease in diffusion layer thickness, and a porous nature due to freeze-drying. At pH 6.8, all formulations from F-1 to F-7 released almost 94–99% of the drug as compared to the F-0 formulation (90%) containing a single polymer.

The addition of a second hydrophilic polymer such as CMC proved fruitful to enhance drug release from βCD-CMC nanosponges. Usually, when two hydrophilic polymers are utilized to fabricate a system, this accelerates its swelling capacity as well as the release of entrapped drug upon contact with aqueous media. Furthermore, F-8 and F-9 formulation depicted a limited drug release due to the increased cross-linker concentration, since an increasing cross-linker concentration retains the drug inside the cross-linked system due to the formation of tight junctions and a dense network [52,77,78]. Song et al. fabricated carboxymethyl-β-cyclodextrin-grafted chitosan nanoparticles as an oral-delivery carrier of protein drugs and reported typical controlled sustained release profiles of 8–12 h [79]. However, in our study, drug-loaded nanosponges displayed initial abrupt drug release profiles within 5 min, which attained equilibrium in 20 min, and almost 99% of the drug was released within 120 min. Thus, the prime objective of this study was achieved, making it a promising drug-delivery system to enhance the aqueous solubility of DTX, which markedly differed from other nanotechnologies. The overall results of our dissolution studies show that the nature (hydrophilic) and quantity of the fundamental reactants used have a profound effect on improving the release profile of a poorly soluble drug (docetaxel).

### 2.12. In Vivo Toxicity Study

#### 2.12.1. Clinical manifestations

Table 3 presented the clinical findings of toxicity studies. No deaths were observed in any groups during the study period. No significant changes in body weight and the food consumption of rats were observed in tested and control groups. There were no clinical signs of ailments related to administration-fabricated nanosponges. Additionally, there were no toxic hazards and no variations in physiological habits of rats in treated groups. Sari et al. conducted a toxicity study and reported no mortality of subjects at the end of study, in accordance with our findings [80]. Onwusonye et al. also executed an acute oral toxicity study of methanol leaf extracts of Croton Zambesicus in mice and revealed no mortality of animals, as in our findings [81].

#### 2.12.2. Blood Analysis

A complete hematological analysis was performed to examine the polymeric network’s effect on the biological system including TLC, hemoglobin, neutrophils, lymphocytes, eosinophils, monocytes, TRBC, platelet count, MCV, MCHC. All values were within a normal range, as shown in Table 4, which confirmed the bio-compatibility of the fabricated nanosponges. Ali et al. conducted a toxicity study and found no significant changes in the biochemical analysis of blood [82]. Barkat et al. fabricated a polymeric network for the colon-targeted delivery of oxaliplatin. In this study, rabbits were used as a model animal to carry out toxicity studies, and similar results to our findings were reported [83].

#### 2.12.3. Histopathological Analysis

**Figure 10:**
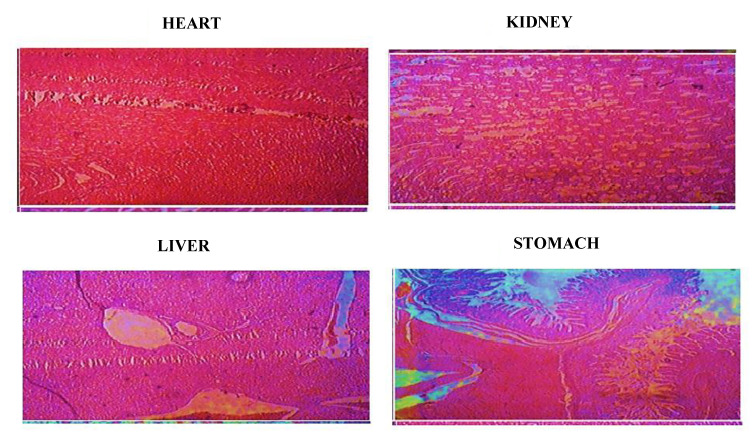
Histopathological micrographs, showing the effect of nanosponges on the cells of epithelial origin of vital organs.

A histopathological analysis was performed to assess the damage posed to organs and tissues by nanosponges [84]. A macroscopic examination of organs depicted no abnormality in rat necropsies. Microscopic examination of tissue slides revealed no signs of lesion, hemorrhage, disruption, deformations, or any type of histopathological change within the vital organs (heart, kindly, liver and stomach), as shown in Figure 10. Shende et al. conducted a toxicological study of β-CD nanosponges in rats of either sex, and reported similar findings [85].

## 3. Conclusions

In this study, a novel oral drug delivery system of docetaxel loaded bi-polymeric nanosponges with enhanced aqueous solubility was successfully formulated using the free-radical polymerization technique. The results of FT-IR, DSC, TGA and PXRD clearly demonstrated the successful cross-linking of the reactants, thermal stability and amorphous nature of a bi-polymeric network. SEM micrographs revealed the porous and spongy structure of the grafted system. The aqueous solubility of docetaxel was considerably enhanced (up to 14-fold), which was attributed to the formation of an inclusion complex with cyclodextrin, as well as the drug’s entrapment in the bi-polymeric matrix. The percent release of DTX can be decreased or increased by utilizing different polymer, monomer and cross-linker concentrations. An acute oral toxicity study further endorsed that the fabricated drug delivery system is non-toxic for a biological system. The consequent of this study suggests that DTX-loaded βCD-CMC-co-poly (AMPS) nanosponges should be regarded as a promising delivery system to enhance the solubility, drug release characteristics and oral bioavailability of DTX in the future.

## 4. Materials and Methods

### 4.1. Materials

Docetaxel was purchased from Xian Wharton Bio-Tech Co., Ltd., Caotan Rd Xi’an, China. Carboxymethyl chitosan (CMC), β-cyclodextrin(βCD), 2-Acrylamido-2-methylpropane sulfonic acid (AMPS), N, N′-Methylene bis(acrylamide) (MBA) and Ammonium per sulphate (APS) were supplied by Sigma-Aldrich, St. Louis, MO, United States of America. All aqueous solutions were prepared with deionized water obtained from Pharmaceutics Research Lab of the Islamia University of Bahawalpur, Bahawalpur, Pakistan.

### 4.2. Methods

#### Fabrication of βCD-CMC g-poly (AMPS) Nanosponges

The free-radical polymerization technique with slight modifications was executed to synthesize bi-polymeric nanosponges. Varying concentrations of polymer, monomer and cross-linker were used during the synthetic process. The composition of initial reaction mixtures used for the fabrication of polymeric nanosponges is given in Table 5 For the preparation of reaction mixture, firstly, a specific quantity of β-CD was dissolved in 70% ethanol with continuous stirring at 50 °C (polymer solution 1). At the same time, carboxymethyl chitosan was dissolved in water to form a clear viscous solution with continuous stirring (polymer solution 2). Polymer solution 1 was added to polymer solution 2 and stirred for 30 s. Secondly, AMPS was dissolved in a specific amount of water and continuously stirred at 30 rpm (monomer solution). APS/SMS solution was prepared and added to AMPS solution with vigorous stirring, followed by the dropwise addition of mixture to bi-polymeric solution. The aqueous solution of cross-linker (MBA) was prepared at 50 °C and dropwise added to reaction mixture. The final solution was immediately transferred to a round-bottom flask and reflux condensation was carried out at 80 °C for 3 h. After refluxing, transparent fluffy mass was sieved to obtain nanosponges of uniform size and dried in oven at 40 °C for the whole night to a constant mass.

### 4.3. Characterization of Fabricated Nanosponges

#### 4.3.1. Fourier Transform Infrared Spectroscopy (FTIR)

Fourier transform infrared spectroscopy (FTIR) is an analytical technique used to endorse the cross-linking reaction required for the successful formulation development of nanosponges. FTIR of polymers (β-CD and CMC), AMPS, DTX, physical mixture and formulated nanosponges were carried out to find the functional groups. Attenuated total reflectance (ATR) Bruker FTIR (Tensor 27 series; Bruker Co., Bremen, Germany) was used. FTIR spectrum was obtained in the range of 4000–650 cm^−1^.

#### 4.3.2. Thermo-Gravimetric Analysis (TGA)

For thermo-gravimetric analysis (TGA), Universal V4.5A TA Instrument SDT Q600 series was used (New Castle, DE, USA). Small quantities (0.5–3 mg) of pure ingredients along with tested formulations were placed in an open platinum pan attached to a microbalance. Samples were heated at 20 °C/min from 25–550 °C under a nitrogen purge. All the measurements were made in triplicate.

#### 4.3.3. Differential Scanning Calorimeter (DSC)

For differential scanning calorimeter (DSC), Universal V4.5A TA Instrument SDT Q600 series (New Castle, DE, USA) was used to determine the glass transition temperature of samples. DSC analysis of βCD, CMC, AMPS and blank tested formulations were performed by placing the small quantities (0.5–3 mg) of these samples in a standard aluminum pan. Temperature range was kept at 25–550 °C and heating rate was adjusted at 20 °C/min under a nitrogen purge. All samples were analyzed in triplicate.

#### 4.3.4. Powder X-ray Diffraction (PXRD Analysis

Powder X-ray Diffraction (PXRD) analysis of pure β-cyclodextrin, carboxymethyl chitosan, docetaxel, physical mixture and tested formulations were carried out using X-ray diffractometer JEOL, Tokyo, Japan (Model JDX-3532). All these powdered samples were tightly packed in an aluminum cell, which was exposed to CuKa monochromatic radiations of wavelength 1.54056 A°. The scanning regions of diffraction angle 2θ were 5°–60° at a scan rate of 3 °/min.

#### 4.3.5. Particle Size Analysis

Particle size analysis was carried out by Zetasizer (Malvern Instruments, Worcestershire, UK). Nanosponges were suspended with filtered ultra-pure water and placed in a clear disposable zeta cell for each measurement [60,86]. The selected and appropriate cuvette cell was filled with the prepared sample and all the air bubbles were removed. Hydrodynamic diameter and polydispersity index were determined.

#### 4.3.6. Scanning Electron Microscopy

Scanning electron microscopy (SEM) was executed to observe the surface morphology and shape of nanosponges under scanning electron microscope (JSM5910, JEOL, Tokyo, Japan). For SEM measurement, the formulated preparations of nanosponges were fixed on metal stubs using double-sided adhesive tape. A gold coating of 300 A on stub was carried out under gold sputter and analyzed under high-resolution scanning electron microscope using various magnifications [87].

#### 4.3.7. Solubilization Efficiency

The solubilization efficiency of all formulations was evaluated in distilled water and buffer solutions of pH 1.2 and pH 6.8. A specific quantity of docetaxel (5 mg) was added in 10 mL of all above solutions. Weighed quantity of tested formulation (25 mg) was also suspended into different solutions containing an equal amount (5 mg) of docetaxel separately. All suspensions were stirred for 24 h using a mechanical shaker (MSC-100, Ningbo Hinotek Technology Co., Ltd., Zhejiang, China) at 25 ± 1 °C. Each suspension was centrifuged at 20,000× *g* rpm for 10 min using centrifuge machine (Model 4000, Hunan Cenlee Scientific Instruments Co., Ltd. Changsha, Hunan, China). The supernatant was collected, filtered, diluted and analyzed on UV-Visible Spectrophotometer at 230 nm wavelength in order to determine the solubility enhancement of docetaxel by prepared formulations [60].

#### 4.3.8. Sol Gel Analysis

Sol-gel analysis was performed to investigate the uncross-linked polymer fraction in gel structure. For this purpose, 500 mg of formulation was subjected to Soxhlet extraction in round bottom flask with 50% ethanol for 4 h at 85–90 °C. After a specified time interval, the water-insoluble part of nanosponges was removed from solvent, placed in labeled petri dishes and dried in vacuum oven at 40 °C for 24–72 h to a constant weight [53]. For the determination of sol and gel% of nanosponges, the following equations were used

(1) $$\mathrm{Sol}\;\%=\frac{W1-W2}{W1}\times 100$$

Gel % = 100 − sol%(2)

where W_1_ is initial weight of formulation before extraction process and W_2_ is the final weight of formulation after the extraction process.

#### 4.3.9. Swelling Studies

The dynamic swelling of all developed formulations was carried out by using dialysis membrane. A total of 500 mg of nanosponges were accurately weighed, sealed in dialysis membrane, immersed in their respective pH solutions (1.2, 4.5 and 6.8), allowed to swell, and then removed after a predefined time interval of 0, 5, 10, 15, 20, 30, 45, 60, 90, 120 min. Their surfaces were cleaned by Whatmann’s filter paper, hanged until no drop was oozing and again immersed in solution. For the measurement of dynamic swelling ratio (q), the following formula was used [40].

(3) $$q=\frac{W_{S}}{W_{D}}$$

where W_S_ is swollen nanosponges’ weight at time t and W_D_ is the initial dried weight of nanosponges.

#### 4.3.10. Drug Loading Studies

**Figure 11:**
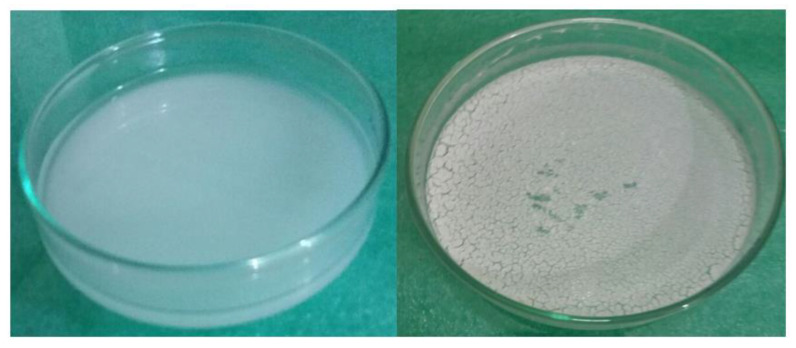
Drug loading of synthesized nanosponges before and after lyophilization.

The loading of a drug (DTX) was carried out by freeze-drying technique. A total of 1% w/v drug solution was prepared in a solvent mixture of methanol and water at 4:1 ratio. The drug solution was then poured on a weighed amount of nanosponges in a Petri-dish and sonicated for 10–15 min. The ratio of drug to nanosponges was 1:10. After sonication, particles were then allowed to swell in drug solution for 1 h at room temperature. Finally, they were lyophilized for 8 h. The dried particles were recovered and stored at 25 °C. Figure 11 represents the drug loading before and after lyophilization.

#### 4.3.11. Percent Drug-Loaded Contents (%DLC) and Percent Drug Entrapment Efficiency (%DEE)

Drug-loading content (%DLC) and drug entrapment efficiency (%DEE) of all grafted polymeric nanosponges were assessed by the absorption and extraction technique. In this study, firstly, the DTX-loaded nanosponges were accurately weighted and transferred into a 5 mL solution of pH 6.8. Finally, the resultant solution was stirred at 100 rpm for 1 h, filtered through 0.45-μm cellulose membrane filter and analyzed by UV spectrophotometer at 230 nm. The DEE (%) and DLC of polymeric nanosponges were calculated using the following formula:

(4) $$\mathrm{Drug}\;\mathrm{Entrapment}\;\mathrm{Efficiency}\;(\%)=\frac{\mathrm{Actual}\;\mathrm{drug}\;\mathrm{content}\;\mathrm{in}\;\mathrm{nanosponges}}{\mathrm{Theoretical}\;\mathrm{drug}\;\mathrm{contents}\;\mathrm{in}\;\mathrm{nanosponges}}\times 100$$

(5) $$\mathrm{Drug}\;\mathrm{Loaded}\;\mathrm{Contents}\;(\%)=\frac{\mathrm{Weight}\;\mathrm{of}\;\mathrm{entrapped}\;\mathrm{drug}\;\mathrm{in}\;\mathrm{nanosponges}\;\left(\mathrm{mg}\right)}{\mathrm{Weight}\;\mathrm{of}\;\mathrm{drug}\;\mathrm{loaded}\;\mathrm{nanosponges}\left(\mathrm{mg}\right)}\times 100$$

#### 4.3.12. In Vitro Drug Release Studies

The release profile of drug (DTX) from fabricated nanosponges was determined by USP dissolution apparatus II operated at 37 ± 2 °C temperature with 50 rpm. Weighed quantity of drug loaded formulations (100 mg) were enclosed in dialysis membrane and suspended in 500 mL of dissolution media of pH 1.2, pH 4.5 and pH 6.8. At pre-determined time intervals, samples were withdrawn and analyzed by a UV-visible spectrophotometer (Memmert, Schwabach, Germany) at 230 nm to assess the percent drug (docetaxel) release. All measurements were made in triplicate.

#### 4.3.13. In Vivo Toxicity Studies

An in vivo toxicity study was conducted on 12 healthy adult male Wistar rats according to the ARRIVE guidelines. The study protocols were reviewed and approved on 1 July 2020 by the institutional ethics committee “Pharmacy Animal Ethics Committee (PAEC)” under reference number PAEC/2020/31, The Islamia University of Bahawalpur, Punjab-Pakistan. One week before the study started, Wistar rats were acclimated in the quarantine area. All animals were marked and housed in wooden cages in two groups at 25 °C under standard laboratory conditions for a period of 14 days. Group I were defined as the control group and given free access to food and water. Group II was treated with the suspension of fabricated nanosponges of 5 g/kg of body weight using nasogastric tube. These animals were keenly physically discerned for general conditions such as the consumption of food and water, body weight, convulsions, mortality rate, hyperactivity, grooming, urination, lacrimation, salivation, pain response, touch response, corneal reflex, righting reflex, writhing reflex and gripping strength during the whole experimentation period. On the 15th day of study, blood samples were collected for hematological examination. Furthermore, all the rats were slaughtered. Different organs were collected and sent to the pathology laboratory to investigate toxic effects in the histopathological examination of organs.

## Figures and Tables

**Table 1:** Average particle size of the developed nanosponges.

S/No	Formulation Code	Average Particle Size of Nanosponges (nm)
01	F-0	195 ± 3
02	F-1	213 ± 2
03	F-2	238 ± 5
04	F-3	250 ± 4
05	F-4	224 ± 3
06	F-5	209 ± 4
07	F-6	211 ± 4
08	F-7	218 ± 3
09	F-8	205 ± 4
10	F-9	199 ± 5

**Table 2:** %DEE, %DLC, sol and %gel fraction of all synthesized nanosponges.

Formulation Code	Varying Component	Entrapment Efficiency %	Drug Loaded Contents %	Sol Fraction %	Gel Fraction %
F-0	Without CMC	75.33	82.70	14.64	85.36
F-1	β-CD & CMC	79.26	83.67	9.56	90.44
F-2		83.10	87.58	7.06	92.94
F-3		87.41	91.74	4.46	94.54
F-4	AMPS	78.64	86.32	13.80	86.20
F-5		81.74	88.92	9.56	90.44
F-6		85.32	89.32	4.26	93.74
F-7	MBA	76.73	86.57	11.76	88.24
F-8		74.44	83.62	9.56	90.44
F-9		71.40	80.43	7.50	95.11

βCD-β cyclodextrin, CMC-Carboxymethyl chitosan, AMPS- 2-Acrylamido-2-methylpropane sulfonic acid and MBA—Methylene bis-acrylamide.

**Table 3:** Clinical findings of an acute oral toxicity study of developed nanosponges.

Observations	Group I (Control)	Group II (Nanosponges Treated)
Signs of illness	Nil	Nil
Body weight (g)		
Pretreatment	233 ± 1.06	234 ± 1.03
Day 1	233 ± 1.07	232 ± 0.63
Day 7	234 ± 0.92	233 ± 0.57
Day 14	235 ± 1.05	234 ± 1.03
Water intake (mL)		
Pretreatment	37.40 ± 2.05	40.50 ± 2.15
Day 1	39.31 ± 1.10	38.66 ± 1.40
Day 7	36.13 ± 2.04	41.21 ± 2.52
Day 14	38.97 ± 1.71	39.24 ± 1.31
Food intake (g)		
Pretreatment	15 ± 1.51	17 ± 1.24
Day 1	17 ± 1.01	16 ± 1.01
Day 7	16 ± 1.61	18 ± 1.51
Day 14	16 ± 1.21	17 ± 2.01
Ocular Toxicity	Not observed	Not observed
Simple irritation	Not observed	Not observed
Dermal toxicity	Not observed	Not observed
Mortality rate	Not observed	Not observed

Note. All values are expressed as mean ± SD (*n* = 3).

**Table 4:** Blood analysis of test animals in control group I and test group II.

Parameters/Tests	Group I (Control)	Group II (Treated with Nanosponges)
Hemoglobin (g/dL)	13.51 ± 0.39	13.38 ± 0.40
Red blood cells × 10^6^/mm^3^	5.16 ± 0.41	5.36 ± 0.51
White blood cells × 10^9^/L	6.89 ± 0.12	6.71 ± 0.30
Platelets × 10^9^/L	4.11 ± 2.01	4.16 ± 2.02
Neutrophils (%)	55.40 ± 3.95	57.11 ± 2.11
Lymphocytes (%)	38.50 ± 2.08	41.21 ± 1.92
Monocytes (%)	3.60 ± 0.31	3.40 ± 0.41
Mean corpuscular volume (%)	83.00 ± 2.30	82.04 ± 2.50
Mean corpuscular hemoglobin pg/cells	23 ± 2.75	25 ± 2.13
Mean corpuscular hemoglobin concentration (%)	35.10 ± 1.21	33.21 ± 2.41

Note. All values are expressed as mean ± SD (*n* = 3).

**Table 5:** Composition of βCD-CMC-g-poly (AMPS) Nanosponges.

Formulation Code	βCD (g/100 g)	CMC (g/100 g)	AMPS (g/100 g)	MBA (g/100 g)	APS/SMS (g/100 g)
F-0	2	-	24	8	0.8/0.8
F-1	0.4	0.4	24	8	0.8/0.8
F-2	0.8	0.8	24	8	0.8/0.8
F-3	1.2	1.2	24	8	0.8/0.8
F-4	0.4	0.4	16	8	0.8/0.8
F-5	0.4	0.4	24	8	0.8/08
F-6	0.4	0.4	32	8	0.8/0.8
F-7	0.4	0.4	24	6	0.8/0.8
F-8	0.4	0.4	24	8	0.8/0.8
F-9	0.4	0.4	24	10	0.8/0.8

βCD-β cyclodextrin, CMC-Carboxymethyl chitosan, AMPS-2-Acrylamido-2-methylpropane sulfonic acid, APS-Ammonium per sulphate, SMS—Sodium metabisulphite, MBA—Methylene bis-acrylamide.

## Data Availability

Not applicable.
